# “We are called the et cetera”: experiences of the poor with health financing reforms that target them in Kenya

**DOI:** 10.1186/s12939-019-1006-2

**Published:** 2019-06-24

**Authors:** Evelyn Kabia, Rahab Mbau, Robinson Oyando, Clement Oduor, Godfrey Bigogo, Sammy Khagayi, Edwine Barasa

**Affiliations:** 1 Health Economics Research Unit, KEMRI-Wellcome Trust Research Programme, P.O. Box 43640-00100, Nairobi, Kenya; 2 African Population and Health Research Centre, Nairobi, Kenya; 3 KEMRI-Centre for Global Health Research, Kisumu, Kenya; 4 Nuffield Department of Medicine, University of Oxford, Oxford, UK

**Keywords:** Experiences, The poor, Health financing reforms, Kenya

## Abstract

**Background:**

Through a number of healthcare reforms, Kenya has demonstrated its intention to extend financial risk protection and service coverage for poor and vulnerable groups. These reforms include the provision of free maternity services, user-fee removal in public primary health facilities and a health insurance subsidy programme (HISP) for the poor. However, the available evidence points to inequity and the likelihood that the poor will still be left behind with regards to financial risk protection and service coverage. This study examined the experiences of the poor with health financing reforms that target them.

**Methods:**

We conducted a qualitative cross-sectional study in two purposively selected counties in Kenya. We collected data through focus group discussions (*n* = 8) and in-depth interviews (*n* = 30) with people in the lowest wealth quintile residing in the health and demographic surveillance systems, and HISP beneficiaries. We analyzed the data using a framework approach focusing on four healthcare access dimensions; geographical accessibility, affordability, availability, and acceptability.

**Results:**

Health financing reforms reduced financial barriers and improved access to health services for the poor in the study counties. However, various access barriers limited the extent to which they benefited from these reforms. Long distances, lack of public transport, poor condition of the roads and high transport costs especially in rural areas limited access to health facilities. Continued charging of user fees despite their abolition, delayed insurance reimbursements to health facilities that HISP beneficiaries were seeking care from, and informal fees exposed the poor to out of pocket payments. Stock-outs of medicine and other medical supplies, dysfunctional medical equipment, shortage of healthcare workers, and frequent strikes adversely affected the availability of health services. Acceptability of care was further limited by discrimination by healthcare workers and ineffective grievance redress mechanisms which led to a feeling of disempowerment among the poor.

**Conclusions:**

Pro-poor health financing reforms improved access to care for the poor to some extent. However, to enhance the effectiveness of pro-poor reforms and to ensure that the poor in Kenya benefit fully from them, there is a need to address barriers to healthcare seeking across all access dimensions.

## Background

Health financing reforms comprise a crucial part of the development of the health sector in low and middle-income countries (LMICs) [1]. Ongoing global debates have been advocating for health systems to transition from high dependency on out of pocket payments (OOPs) towards prepayment arrangements that enhance financial risk protection for the poor [1]. The Kenyan constitution stipulates that everyone has the right to the highest achievable standard of health, which entails the right to health services. In addition, Kenya has made a commitment to reform its health financing system to achieve universal health coverage (UHC) by the year 2022 [2]. The goal of UHC is to ensure that everyone can use the health services they need without the risk of impoverishment [3].

Kenya’s health financing system provides coverage to the population through a mix of contributory health insurance, supply-side subsidies financed through general taxation, and OOPs by healthcare consumers [4, 5]. In 2015/16, government health expenditure accounted for 6.7% of the total government expenditure and total health expenditure (THE) per capita was US$ 78.6 [6]. Current health expenditure as a percentage of the gross domestic product was 5.2% in 2015 while in other African countries it ranged from 2.5% in South Sudan to 18.3% in Sierra Leonne [7]. Public finances, private finances, and donors contributed 37, 39.6 and 23.4% of the THE respectively, with household OOPs contributing 26.1% of the THE [6].

Through a number of policy reforms, Kenya has demonstrated its intent to extend health financing coverage for poor and vulnerable groups. These reforms include the provision of free maternity services in all public health facilities from 2013 in an effort to reduce the high maternal mortality rate (362 maternal deaths per 100,000 live births as of 2014) and to improve access to facility-based deliveries [8–11]. Second, since healthcare costs were a key barrier to access especially among the poor, user-fee removal in public primary healthcare (PHC) facilities was introduced from 2013 to improve access and utilization of health services. Previously, user fees entailed payment of KES 10 (US$ 0.1) at dispensaries and KES 20 (US$ 0.2) at health centers under the 10/20 policy which had been introduced in 2004 [8–10, 12]. Third, a health insurance subsidy programme for the poor (HISP) was piloted from April 2014 and scaled up from August 2016 with the aim of reducing OOPs, increasing access and utilization of health services and ultimately improving health outcomes of the poor [13]. Under HISP, the National Hospital Insurance Fund premiums for the poorest households are fully subsidized by the government and through donor support [13]. HISP beneficiaries access outpatient and inpatient care at NHIF accredited public, faith-based and low-cost private health facilities [13].

While these are important developments in Kenya’s health financing system, empirical evidence from LMICs reveals that public spending on health mainly benefits the rich rather than the poor for whom services are intended [14–18]. In addition, replacing direct payments for healthcare with prepayment mechanisms does not guarantee access to care for the poor since direct costs for care only account for a small part of the total financial costs especially at government health facilities; financial costs represent only one of the access barriers [3]. The poor and vulnerable are therefore more predisposed to healthcare spending that is catastrophic and that pushes them deeper into poverty [15, 17, 19]. This challenge is greater in LMICs because of the large proportion of poor populations, constrained fiscal space and weak institutional and organizational capacities [20, 21].

In Kenya, the available evidence points to inequity and the likelihood that the poor will be left behind along both dimensions of UHC (financial risk protection and service coverage). While the incidence of catastrophic healthcare expenditure is 2 % among individuals in the richest quintile, those in the poorest quintile have a catastrophic health expenditure incidence of 10% [4]. Additionally, 62% of Kenyans in the richest quintile have effective coverage with priority maternal and child health interventions while effective coverage among those in the poorest quintile is only 37% [22]. Following the removal of user fees for deliveries in Kenya, there was increased uptake of facility-based deliveries among women living in poverty [23]. However, a study exploring patients’ experiences during delivery after the introduction of free maternity services showed that women reported being neglected during delivery or labour and being physically and verbally abused, with the latter being common against women of lower socio-economic status [24]. In terms of health insurance coverage, 39% of Kenyans in the richest quintile have health insurance compared to 3 % for those in the poorest quintile [25]. HISP aims to extend insurance coverage to poor and vulnerable groups and evaluations of the pilot program showed that HISP enabled beneficiaries to access health services at no cost. However, some of the challenges reported include; low level of awareness among beneficiaries about service entitlements which led to some healthcare providers taking advantage of beneficiaries’ ignorance by charging them additional fees, absence of some health services such as imaging and drugs and lack of grievance redress mechanisms within the NHIF. Most of the NHIF accredited facilities where beneficiaries were capitated to were also reported to be located mainly in urban areas which led to high transport costs [26].

The poor have the highest disease burden [27], reduced access to healthcare services across all access dimensions [16] and many of them may completely fail to utilize health services [28]. “Access is related to the timely use of services according to need” [16] and barriers to accessing health services can stem from the demand side and/or the supply side [16]. Demand-side factors influence an individual’s, household’s or community’s ability to utilize healthcare services while supply-side factors are health systems factors that prevent individuals, households or communities from utilizing health services [27].

It is evident that inequities exist despite the existence of health financing reforms that target the poor and other vulnerable groups in Kenya. This study examined the experiences of the poor with health financing reforms that target them and the challenges they face in benefiting from these reforms in Kenya. Focusing only on the experiences of the poor distinguishes this study from other studies that have explored the experiences of the general population.

## Methods

### Study setting

The Kenyan government is made up of a national government and 47 county governments, with the latter tasked with the delivery of health services to their citizenry [29]. Kenya’s health system is organized into 4 tiers: Tier 1/community which is comprised of community units whose primary aim is creating demand for health services, Tier 2/primary care which is made up of health centers, dispensaries, and clinics, Tier 3/secondary referral which is made up of county hospitals and Tier 4/tertiary referral which is made up of national referral hospitals [29].

In 2015, the health workforce density (doctors, clinical officers and nurses/midwives) in the country was 13.8 per 10,000 population [30] while the WHO recommends 22.8 skilled health workers per 10,000 population [31]. With regards to access to roads nationally, 39.1, 19.8, and 7.7% households were located five kilometers (km) or more, 10 km or more and 20 km or more from a paved road respectively in 2014 [32].

We conducted the study in two counties in Kenya, Nairobi, and Siaya. The counties were purposively selected to include an urban (Nairobi) and rural (Siaya) county with an established health and demographic surveillance system (HDSS) and the HDSS that had collected the most recent socio-economic data of the residents. There are seven HDSS in Kenya and they are located in six counties: the Nairobi Urban HDSS (NUHDSS) and Kibera HDSS both in Nairobi County, Kenya Medical Research Institute-Center for Disease Control and Prevention (KEMRI/CDC) HDSS, Kilifi HDSS, Kombewa HDSS, Webuye HDSS and Rusinga HDSS which are located in Siaya, Kilifi, Kisumu, Webuye and Homabay counties respectively. HDSS collect key demographic data such as births and deaths in addition to socio-economic data in a geographically demarcated population over time. Counties with HDSS were crucial for this study to facilitate sampling of people from households in the lowest wealth quintile since they were the target study population.

The two HDSS purposively sampled in this study were the NUDHSS which was established in 2002 and is run by the African Population and Health Research Centre and the KEMRI/CDC HDSS which was established in 2001. The NUDHSS covers two informal settlements; Viwandani and Korogocho [33] and these informal settlements are located in two out of the 17 sub-counties in Nairobi County, that is, Makadara and Ruaraka sub-counties respectively. Viwandani is the most populated with most of the residents working in nearby industries and, the population is also highly mobile compared to the Korogocho population [33]. There were 25,793 households, with a total population of 70,000 individuals in the NUHDSS by 2013 [33]. Data on key health and demographic indicators such as births, deaths and migration status are collected three times in a year while data on household characteristics such as housing conditions, the source of livelihood and possessions are collected once every year [33]. The KEMRI/CDC HDSS is located in three sub-counties, Rarieda, Siaya, and Gem, out of the six sub-counties in Siaya County [34, 35]. The KEMRI/CDC HDSS had 63,943 households, and a population of 255,000 as of 2015 [35, 36]. The population is mainly rural and the main sources of livelihood are subsistence farming, fishing and small-scale trading [36]. Two rounds of data collection on key health and demographic indicators are conducted each year within the KEMRI/CDC HDSS [35, 36] while data on the socio-economic status of the households are collected biennially [36]. Table 1 presents the demographic and health indicators of the two study counties.

**Table 1:** Key demographic and health indicators in the study counties

Indicator	Nairobi County (urban)	Siaya County (rural)	Kenya
Population in 2015/16 [37]			
Total	4,463,000	985,000	45,371,000
Male	2,237,000 (50.1%)	466,000 (47.3%)	22,393,000 (49.4%)
Female	2,226,000 (49.9%)	519,000 (52.7%)	22,977,000 (50.6%)
Poverty rate in 2015/16 [38]	16.7%	33.8%	36.1%
HDSS [33, 35, 39–44]			
Population	70,000	255,000	> 868,472
Households	25,793	63,943	> 173,220
Health facilities in 2015 [45, 46]			
Public	161	123	4929
Non-governmental	118	7	347
Faith-based	100	16	1081
Private	543	28	3797
Utilization of traditional/faith healers/herbalists	0.9%	0.9%	0.8%
Health personnel in public facilities in 2015 [45, 46]			
Nurses (per 100,000 people)	53	33	55
Doctors (per 100,000 people)	14	2	10
Clinical Officers (per 100,000 people)	6	25	21
Access to health services [37]			
Utilization of curative services	17.3%	30.6%	18.0%
Utilization of promotive/preventive services	2.4%	7.3%	4.2%
Health facility deliveries	90.8%	83.9%	67.2%
Measles I & II immunization coverage (0–59 months)	77.7%	83.4%	77.4%
Health financing			
Total government health spending, 2014/15 (per capita in US$) [47]	34.9	76.8	66.7
Health insurance coverage (2015/2016) [37]	40.7%	7.6%	19.0%

### Study design and data collection

We conducted a qualitative cross-sectional study where we interviewed HISP beneficiaries and people in the lowest wealth quintile residing in the HDSS. HDSS collect data on household assets and use it to develop an asset index that ranks households into wealth quintiles each comprising 20% of the population. Quintile 1 representing the poorest, and quintile 5 representing the richest households [34]. A list of people already identified to be in the poorest quintile following the most recent round of data collection at both HDSS was used to identify potential study participants.

We also collected data from individuals enrolled in HISP (HISP beneficiaries). HISP beneficiaries are members from households in the poorest quintile and these households also have orphans and vulnerable children or elderly persons above 65 years of age or people with severe disabilities [8]. HISP beneficiaries benefit from the government’s cash transfer program and they are selected from the government’s social assistance programs registry for the poor which is managed by the Ministry of Labour, Social Security, and Services. The government uses proxy-means testing and community verification to identify poor people who are eligible to benefit from social assistance programs [13].

In each county, we obtained a list of individuals in the lowest wealth quintile in the HDSS and a list of HISP beneficiaries. Study participants identified from the HDSS were not HISP beneficiaries but some HISP beneficiaries were residing in the HDSS. Maximum variation sampling, one of the types of purposive sampling, was employed in the selection of both focus group discussion (FGD) and in-depth interview (IDI) participants in order to capture diverse insights about the research topic [48, 49].

**Fig. 1:**
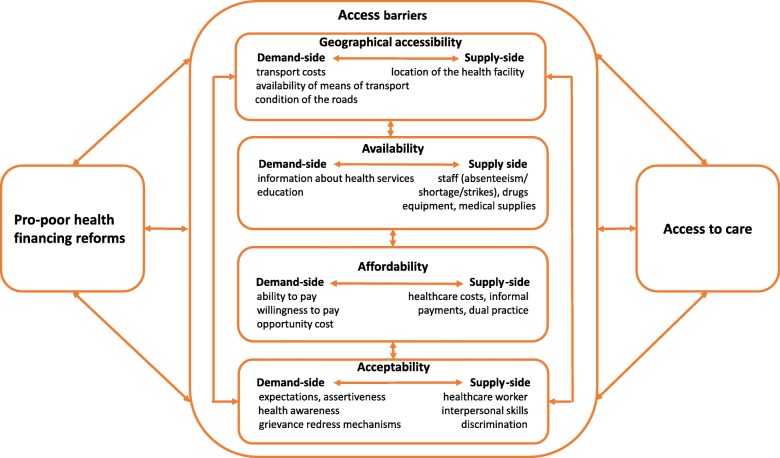
conceptual framework [27]

IDIs were conducted using semi-structured interview guides while discussion guides were used to facilitate the FGDs. The questions in the interview and discussion guides were developed with reference to the study’s conceptual framework (Fig. 1) and they focused on awareness and experiences with health financing reforms with regards to access dimensions: geographical accessibility, availability, affordability and acceptability, benefits and barriers to utilization of health services provided through the health financing reforms. We mobilized and interviewed study participants from central venues within the respective study communities such as chief camps and community resource centers. Written informed consent was obtained from all study participants prior to conducting the interviews and all interviews were audio-recorded with the participants’ consent. The interviews were conducted in Swahili or Dholuo, the languages mainly used in Nairobi and Siaya County respectively, and the audio recordings were augmented by field notes. We conducted 8 FGDs, each lasting between 90 to 150 min and 30 IDIs, each lasting between 30 min to 90 min. Of these 30 IDIs, there were more female than male participants because 10 IDIs were conducted with women with disabilities living in poverty in the two study counties to explore how gender, disability, and poverty intersect to influence how they benefit from health financing reforms; as an additional aim of this study. These women with disabilities living in poverty were women in the lowest quintile residing in the HDSS and HISP beneficiaries and they had been identified through similar purposive sampling procedures. The findings from the additional I0 IDIs have been published in a different paper [50] but these IDIs were included in the data analysis because they also focused on experiences of the poor when accessing health services provided through pro-poor health financing reforms.

Debrief sessions were held between the three authors involved in data collection (RM, RO, and EK) at the end of each day’s data collection. The debrief sessions informed how to improve the interviewing process and together with the field notes, they aided in revision of some questions in the interview guides to enhance clarity and inclusion of emerging themes that needed to be explored further. Data collection was discontinued when data saturation was achieved. We collected data between September and December 2017.

### Conceptual framework

We developed the study’s conceptual framework based on Jacob’s et al., 2011 summary of supply and demand side access barriers across the four access dimensions (geographical accessibility, availability, affordability, and acceptability) [27]. Our framework postulates that pro-poor health financing reforms reduce both supply-side and demand-side access barriers across the four dimensions of access. Under each access dimension, access barriers include: geographical accessibility (location of the health facility, means and cost of transport); availability (drugs, equipment, healthcare workers, information on health services); affordability (service costs, informal payments, clients’ ability and willingness to pay); acceptability (healthcare worker interpersonal skills, individuals expectations, patients assertiveness and level of awareness about health services). Presence or absence of these access barriers is based on the effectiveness of pro-poor health financing reforms which in turn determine individuals, households, and communities’ experiences when accessing healthcare services. In addition, access barriers do not act independently at all times and often, interactions occur across various access barriers [27] to influence peoples experiences when seeking care.

### Data analysis

We analyzed the data using a framework approach which facilitates analysis of data into various themes, comparison of the themes across various transcripts and summarization of data in a systematic manner [51]. We first transcribed all the audio recordings verbatim in Swahili or Dholuo and then translated them into English. We familiarised ourselves with the data by reading and rereading the transcripts and field notes and checked for correctness by re-listening to sections of the audio recordings. The validated transcripts were then imported to NVivo 10 (QSR International) for line by line coding guided by the study’s conceptual framework. This initially entailed open coding where data could be allocated to more than one code. One researcher coded the first few transcripts, identified the key themes and developed the initial analytical framework by building upon the predetermined study’s conceptual framework. The analytical framework was discussed and revised upon achieving consensus among all the authors. After indexing all the transcripts based on the codes and themes in the final analytical framework, charting which entails summarizing the findings from each transcript based on the various themes was done and illustrative quotes were identified. Data was interpreted by identifying connections between the various themes and using this to gain a better understanding of participant experiences with health financing reforms.

## Results

We interviewed a total of 102 participants through FGDs and IDIs. Eight FGDs were conducted with 72 participants, 33 men, and 39 women while 30 participants, 9 men, and 21 women, took part in IDIs. The FGDs comprised both men and women and had between 7 and 12 participants. The median age of FGD participants was 55.5 years and the age range was 18 to 88 years. The median age of IDI participants was 46 years and the age range was 21 to 88 years. Table 2 provides more details about the distribution of FGD and IDI participants in the study counties by gender and age.

**Table 2:** Participant characteristics

	Male	Female	Total	Median Age	Age range
FGDs					
Nairobi County					
FGD 1 with HDSS participants	6	2	8	29.5	19–61
FGD 2 with HDSS participants	4	5	9	39	23–56
FGD 3 with HISP beneficiaries	2	5	7	40	27–58
FGD 4 with HISP beneficiaries	4	4	8	42	28–54
Total	16	16	32	36.5	19–61
Siaya County					
FGD 5 with HDSS participants	4	4	8	45	18–75
FGD 6 with HDSS participants	4	6	10	34	19–83
FGD 7 with HISP beneficiaries	4	6	10	59	20–88
FGD 8 with HISP beneficiaries	5	7	12	66	30–88
Total	17	23	40	56.5	18–88
Summary of FGD participants	33	39	72	45.5	18–88
IDIs					
Nairobi County					
IDIs with HDSS participants	2	6	8	33.5	25–70
IDIs with HISP beneficiaries	2	5	7	39	29–60
Total	4	11	15	35	25–70
Siaya County					
IDIs with HDSS participants	3	4	7	32	21–57
IDIs with HISP beneficiaries	2	6	8	62	45–88
Total	5	10	15	50	21–88
Summary of IDI participants	9	21	30	46	21–88

*FGD* focus group discussion, *IDI* in-depth interview

The results are presented based on the four access dimensions, geographical accessibility, affordability, availability, and acceptability and brief summaries of the key findings are provided under each access dimension.

### Geographical accessibility

### Some of the health facilities contracted to provide healthcare services under pro-poor health financing policies were inaccessible

Study respondents expressed concerns that long distances to health facilities acted as a barrier to seeking healthcare services, especially in rural areas. For instance, even though some HISP beneficiaries had health insurance cards meant to improve their access to care, the healthcare facilities contracted by the NHIF to provide healthcare services were far from their place of residence. Some women who would benefit from the free maternity services also complained of long distances to health facilities.

*“What can make people not to use this program [free maternity services] and give birth at home without visiting the hospital is that hospitals are far, the poor road networks and lack of means of transport. Because if you get labor pains at night then you can’t make it to hospital. Motorbike riders are not around, taxi drivers are also not available, so you will have to wait until morning and you can give birth before morning or even give birth and die.”* Rural HDSS, FGD 1

*“If I lack means of transport, I walk. I can take around two hours to get to the hospital.”* HISP IDI 1, rural resident

The challenge of long distances was compounded by difficulties in obtaining means of transport to health facilities especially in the rural areas and at night.

*“We don’t even have motorbikes and vehicles around the village. So whether the hospital is close or far you must board a motorbike or a vehicle, especially for serious ailments. So many succumb to their ailments because of late arrival to the hospitals.”* Rural HDSS, FGD 1

*“It’s always a big challenge when one falls very sick at night … You’ll just sit up till morning so that you can get a motorbike to take you to the hospital, and it may be late for treatment. But that depends on God if He so wishes you can survive, but it’s just difficult.”* Rural HDSS, FGD 1

Where means of transport were available, the poor conditions of the roads, especially during the rainy seasons, made some health facilities inaccessible to people living in rural areas.

*“If someone falls sick at night. There are paths that even motorbikes cannot pass.”* Rural HDSS, 1D1 2

*“The government should strive to improve the condition of the roads leading to these hospitals because sometimes it rains heavily and it’s difficult to access the hospitals…Personally, I live near Hospital A but at times it rains so heavily that I prefer to go to Hospital B because it’s easier to get there*.” Rural HDSS, FGD 1

### Affordability

### Health financing policies that targeted the poor reduced financial barriers to accessing care

Respondents reported that the introduction of the free maternity services, user fee removal, and HISP reduced financial access barriers and enabled them to utilize health services they would have previously foregone.

*“Before [ free maternity services] people were fearful of delivering in hospitals because they were afraid that if they go and deliver at the hospital, they could not be discharged because they didn’t have money. Therefore, they could just deliver at home, sometimes they could have complications during delivery and that’s when they would be rushed to the hospital when they are already in a bad state.”* Rural HDSS, FGD 2

*“When we didn't have this card [HISP], it was a burden. You find that someone has little income, you have children, you find that someone would fall sick while in the house and they wouldn’t go to the hospital because getting health services requires money. This [HISP] card has helped many people. People used to suffer and they couldn’t go to the hospital, where would you get the money?”* HISP IDI 4, urban resident

### Continued charging of user fees by some health facilities hindered access to care

Even though policies such as user fee removal, free maternity services, and HISP were meant to eliminate OOPs, some respondents reported that they still had to pay user fees to access certain health services. For example, despite the abolition of user fees in public PHC facilities, some facilities continued to charge user fees for laboratory services and injections among other services.

*“They shouldn’t say these things [health services] are free, there is corruption in Kenya, corruption is everywhere. They say these things are free but when you go there you must pay so there is nothing that is 100% free… They say it’s free but it’s not free, it’s true that they will favor you in terms of the price but it won’t be totally free.”* Urban HDSS, FGD 1

*“We pay when we go to dispensary A. We pay at the laboratory KES 50 (US$ 0.5). We pay for injections, if it’s a child its KES 20 (US$ 0.2) if someone is over five years it's KES 50 (US$0.5).”* Rural HDSS, IDI 5

Some women who were beneficiaries of free maternity services incurred OOPs because they had to purchase basic hospital commodities which ought to have been provided by the health facilities but they were not available.

“*If you take an expectant woman there are many things they [public health facility] need you to buy. They are things like gloves, if there is any medicine that you require, you must go and buy it. When you are discharged they send you to the cashier to go and pay. But it is said to be free therefore, we clearly don’t understand how free it is.”* Rural HDSS, FGD 2

*“They don’t pay anything as long as you come with something like a basin, sometimes there is no cotton wool and you are told to come with that too, together with soap and food…Yes! there is no food.”* Urban HDSS, IDI 1

Some HISP beneficiaries reported that they were required to make OOPs because NHIF payments to the health facilities they were capitated to were often delayed.

*“We were tricked; they have wasted our time by giving us invalid cards. I have to use my own money to pay for treatment, so what is the use of having the card? Why should the government lie to me and my family? It is very bad. I always feel deceived when I use my own money to pay for treatment.”* HISP FGD 1, Rural resident

*“I was told that the government doesn’t pay so my card [HISP] is not working. That day I just went back home and bought Panadol but God helped me and I got well…I haven’t gone back with that card I just go [to the hospital] if I have money.”* HISP IDI 1, Rural resident

### Informal payments presented a barrier to accessing care

Some respondents reported having to make informal payments to healthcare workers in order to access healthcare services. For example, some respondents paid bribes to skip long queues in health facilities or to get medicines at a subsidized cost.

*“You will just give out the bribe so that you can get treatment but you also know that what you are doing is wrong…. but if that is the system that is there then you will just have to follow suit to get help.”* Rural HDSS, FGD 1

*“Nowadays it is very common [bribes]. Even here in Hospital A if you don’t bribe someone then you can spend so much time in the queue.”* Rural HDSS, FGD 1

*“They ask you "how much do you have? Do you have KES 500 (US$ 5) so that I can to give you some drugs that were reserved for someone else? Then, you tell them that you have KES 300 (US$ 3), you give them the KES 300 (US$ 3) and they give you the already packed and labeled medicine.”* HISP FGD 2, rural resident

### Rural residents faced high transport costs due to long distances to health facilities

Long distances to healthcare facilities especially in rural areas resulted in high transport costs that presented an access barrier to beneficiaries of pro-poor health financing policies. For example, some HISP beneficiaries found it difficult to access the health facilities they had chosen to seek care from due to lack of money to cater for transport costs.

*“When you fall sick, you have that card [HISP], you live far away and you don't have money for transport, that becomes a problem, it's not easy because if you don't have money for transport you can’t get to the hospital…The government is capable; it can give us money for transport.”* HISP IDI 1, rural resident

“*If someone falls sick at home while the dispensaries and hospitals are far, even public vehicle drivers will charge you expensively, and at night it’s even worse. It’s more expensive.”* Rural HDSS FGD 2

### Availability

### Removal of user fees improved access to health services

User fee removal in primary health facilities and HISP enabled the poor to access health services and drugs at no cost.

*“Removal of charges has been helpful, especially us who are poor. When you go there [health facility], you get medicine, you get treated, you get everything for free. You can also get free health education.”* Urban HDSS, IDI 2

*“It has helped me so much [HISP card] because I live with my brother’s children, both parents passed away. So even if a child is sick, when I go to the hospital and I find that they treat them without asking for money, that gives me the strength to carry the child to the hospital instead of sitting with them in the house when they are sick.”* HISP FGD 2, rural resident

Additionally, some respondents perceived healthcare workers, especially those working in government hospitals, to be knowledgeable, well trained and they reported that they provided appropriate referrals for further management.

*“I choose to go to government hospitals because all the healthcare workers are trained and they understand what they are doing. I really prefer going to government compared to those private hospitals.”* Urban HDSS, IDI 3

*“They are knowledgeable and if they are not able to treat your illness, they refer you to a doctor who has more knowledge about your disease.”* Urban HDSS, FGD 2

### Some of the health facilities contracted under pro-poor health financing policies lacked essential health services

Some of the PHC facilities, public hospitals, and private facilities that provided healthcare services to beneficiaries of programs like HISP and free maternity services were characterized by stock-outs of medicine and medical supplies and lack of or broken-down medical equipment.

*“You are only asked to go and buy a syringe and needle [at dispensary A] but they don’t ask for any payment…. or you are told a certain drug is out of stock and you are asked to go and buy at a pharmacy outside the hospital.”* Rural HDSS, FGD 2

*“The government that pays for us [HISP beneficiaries] these services, doesn’t it pay for our medicine?" So you have to buy medicine and you don’t have an income. That's a big problem.”* HISP IDI 1, Urban resident

*“I was tested and they found that I was developing cervical cancer. I was supposed to go for brachytherapy but I was told that the brachytherapy machine at public hospital A was not working. I was told to go to a private hospital A but I couldn’t afford private hospital A so I went to public hospital B and I was told that the nurses are on strike.”* Rural HDSS IDI 4

Shortage of drugs made some study participants to incur OOPs or borrow money to purchase the prescribed drugs and for those who did not have enough money, they failed to buy the drugs or bought incomplete doses. Absence of some health services for example as a result of break-down of medical equipment and the inability to afford similar services at private facilities made some study respondents to forgo care.

*“I didn't have even a cent…I was prescribed for almost four drugs and one was not available so I didn't buy it, I just used the ones they gave me.”* HISP IDI 2, rural resident

*“If you have been prescribed for some drugs, someone can help you with some money which you use to buy at least half the dose if you can’t buy the full dose.”* HISP IDI 5, rural resident

*“There is a problem at the laboratory, the machine keeps breaking down all the time…you are sent to private hospital A. You need money at private hospital A and if you don’t have that money, you fail to go back to the hospital. You were coming here [government health center] because you knew you will receive complete treatment; you came here because you did not have money.”* Urban HDSS, IDI 1

Public health facilities were also characterized by healthcare worker shortage, absenteeism, and frequent strikes and some respondents also complained of long waiting times.

*“Services are free, facilities are, there but the person [healthcare worker] who is supposed to attend to you is not there.”* Urban HDSS, FGD 1

*“People are suffering; people are not getting healthcare. Where will you get healthcare and the doctors and nurses are on strike?”* HISP IDI 1, rural resident

“*In the laboratory, you can spend even five hours before you are given the results. When the results are out, you are already tired and worn out.”* HISP FGD 2, rural resident

Healthcare worker strikes led to some study respondents seeking care at private health facilities while those who could not afford to seek care at private facilities were forced to delay care seeking until service provision resumed at government health facilities.

“*There are no nurses and since they are not available, people are forced to go to private hospitals but many people cannot afford, it’s expensive…. That’s why a disease can cause you harm because you cannot get help anywhere.”* HISP IDI 1, rural resident

*“I am waiting for them [nurses] to go back to work [after the strike] and I will go to public hospital B. Isn’t it lack of money that has made me not to go to private hospital A? If I could afford, I would have been treated a long time ago.”* Rural HDSS, IDI 4

### Acceptability

#### Beneficiaries of pro-poor health financing policies were discriminated against by healthcare workers

Some respondents reported being discriminated against and receiving less attention from healthcare workers because of their low socioeconomic status.

*“They [healthcare workers] are superior, you cannot tell them anything and the living conditions of the people who live around this area [informal settlement], they treat us with contempt.”* Urban HDSS, FGD 1

*“You find that some of these doctors prefer those with high income who can do anything they’re told to do…you find that they don’t pay attention to those with low-income. So doctors are very different. But there are those who are willing to help anyone.”* Rural HDSS, FGD 1

It was reported that some private healthcare providers also discriminated against HISP beneficiaries because they preferred cash-paying patients.

*“They [healthcare workers] are not good because they insult you "you are disturbing us and yet you are paid for [health services] by the government. They insult you because they get a different patient who is paying in cash.”* HISP IDI 1, Urban resident

“*They perceive us as debtors or people with loans that will be paid later. They take a long time before they attend to us or they can even ignore you.”* HISP FGD 1, Rural resident

#### Ineffective complaints and feedback mechanisms led to a feeling of disempowerment and helplessness among the poor

Some respondents were not aware of avenues they could use to raise complains and where those avenues existed such as suggestions boxes, they were perceived to be ineffective*.*

*“I have gone to the hospital and seen a box written “suggestion box” but I didn’t know its purpose.”* Rural HDSS, FGD 2,

*“Whom will you tell and even if you speak up, no action will be taken and there is nowhere to complain. Yes, the suggestion box is there, you will put your suggestion and they [healthcare workers] are the same ones who will go to read them, so they can't incriminate themselves, so there is no impact”* Urban HDSS, FGD 2

In the absence of appropriate grievance redress mechanisms, the poor felt disempowered and at the mercy of the healthcare workers. They felt incapable of raising complaints when they were not satisfied with the quality of care provided.

*“We don’t have the ability to speak and be heard so we just get satisfied with the services we receive…You know there are those who own Kenya and those who live in it so we are called the et cetera. So it’s not easy for people like us to say something and it is heard…we won’t see it being done until they feel that they can do this to help the people but not because we have said…it’s not possible. They will hear but it will remain as it is”* HISP FGD 2, urban resident

*“You feel that if you complain, you will be suggesting that this person [healthcare worker] doesn’t understand their job so you have to allow them to do what they are doing. Even if it’s not pleasing to you, you have to remain silent because it’s like you don’t have a right to complain…people feel that if they complain, the healthcare worker will leave them alone and they will suffer more so they decide not to say anything”* Rural HDSS, IDI 3

## Discussion

Our study revealed that the poor in the study counties in Kenya faced various healthcare access barriers despite the existence of health financing reforms that target them. Some of the health facilities contracted to provide healthcare services under pro-poor health financing policies were inaccessible. This was because of long distances to health facilities, lack of public transport and poor condition of the roads, especially in rural areas. Distances and location of health facilities have been shown to influence utilization of health services [18] and poor regions in developing countries rarely have good road networks despite being crucial in facilitating access to health facilities [16]. Over a decade ago, residents of rural western Kenya, which neighbors one of the study counties, still faced the same challenge of limited access to public transport which resulted to long travel times for patients who had no option but to walk the long distances to health facilities [52]. In addition, clinic attendance by sick children at PHC facilities in rural western Kenya decreased as the distance from their place of residence to the health facility increased. Similarly, in Rwanda, Ghana, Bangladesh and Vietnam, geographical access barriers similar to those reported in our study negatively impacted access to antenatal care services [53]. Access to care for people residing in rural areas, the elderly and the poor is further limited by the pro-urban distribution of health facilities since urban residents have a higher demand for health services and greater ability to afford costs of care [1].

Financial access is deemed the most crucial determinant of access [16]. Study participants reported that removal of user fees for deliveries and at PHC facilities and having a free health insurance cover enabled them to access health services that they would have previously foregone due to lack of money to cater for healthcare costs. Similarly, there was an increase in skilled birth deliveries and reduction of neonatal mortality in 10 sub-Saharan countries following the abolition of user fees for health facility deliveries [54]. However, user fee removal doesn’t eliminate all financial barriers to accessing facility delivery [55]. The latter is evidenced by the minimal effect on the utilization of skilled birth care among the poorest women despite exemption of maternity fees in Ghana for two decades [55] with the place of residence and distance to the health facility reported to be some of the factors influencing the uptake of skilled birth delivery. In rural areas, transport costs are one of the key healthcare expenses [3] and in our study, high transport costs in rural areas due to the long distances to health facilities acted as an access barrier. This supports literature which shows that the poor spend more money and time to access health facilities in remote areas and this costs act as barriers to accessing care [1, 16] and can delay care seeking [3]. In Ghana, high travel costs made women forgo seeking free skilled birth delivery services [56].

Presence of programs and policies is not an assurance that the poor will benefit from them [57]. Our study findings show that the poor in the study counties still incurred OOPs due to the continued charging of user fees despite their abolition in PHC facilities and lack of drugs and basic hospital commodities. These OOPs also comprised informal payments/bribes to healthcare providers to enable the poor to skip queues and to get drugs at a subsidized cost. OOPs are a regressive form of healthcare financing [16, 17, 57] and for the poor, even minimal healthcare costs resulting from stock-outs of drugs and other hospital supplies are deemed to be unaffordable [15]. In 2015 some patients still reported paying for registration, medicines, injections and laboratory services despite the abolition of user fees in PHC facilities in Kenya [58]. Similarly, despite the existence of free maternity services in Nepal and rural Tanzania, many women reported incurring costs for a facility-based delivery [59, 60]. These entailed transport costs [59], informal payments [59], costs for medicine, consumables, food, and drinks during the hospital stay [60]. Informal payments are predominant among the poorest [61], and this is attributed to poor/rich disparities in supply-side factors such as long waiting times, healthcare provider absenteeism and absence of drugs [62]. Some HISP beneficiaries also incurred OOPs because of delayed payments to the health facilities they had chosen to seek care from. Similarly, the National Health Insurance Scheme in Ghana faced challenges such as delayed reimbursements to healthcare providers offering care to the poor [57].

Access to quality healthcare services relies on the availability of medicines, medical equipment, and basic health facility infrastructure [58]. In this study, stock-outs of medicines, medical supplies and commodities and lack of or dysfunctional medical equipment limited availability of health services provided under pro-poor health financing policies and also led to study participants incurring OOPs to purchase drugs and medical supplies that should have been provided for free. Similarly, user fee exemption policies led to shortage of drugs in Madagascar, South Africa, and Kenya [63] and this emphasizes the need for increased financial commitment to meet the increased demand for services and to reimburse facilities for revenue lost from the removal of user fees [64]. Other factors that influenced participants access to care included healthcare worker shortage relative to the large number of patients seeking health services, staff absenteeism, frequent strikes and long waiting times. Long waiting times are considered to be an indicator of staff and equipment distribution that is not at per with service demand [27]. Kenya’s public health sector is also prone to frequent health worker strikes and this led to 250 days of both nurses or doctors strikes in 2016/17 and this negatively affected access to both inpatient and outpatient services [65].

Acceptability is an access dimension that is often forgotten [66] despite the fact that healthcare workers conduct is considered an essential component in the provision of quality healthcare [67] and patients also value respectful treatment from healthcare workers [17]. Power differences between healthcare providers and patients are common, with the former having more power in most cases. This influences healthcare providers views about the roles of service users in providing oversight over health services and this subsequently influences how they respond to social accountability mechanisms [68]. The poor in the study counties felt they were discriminated against by healthcare workers because of their low socio-economic status. Some study participants reported they received less attention from healthcare workers because they lived in informal settlements, and because they were beneficiaries of free healthcare from government programs that targeted the poor. Beneficiaries of pro-poor health financing reforms in the study counties also considered grievance redress mechanisms to be ineffective. They were unable to raise complaints and they felt voiceless because of their low socio-economic status. Our findings are consistent with literature which suggests that the poor lack the power to demand for their rights and they are therefore prone to abuse and discrimination by health service providers [55]. Similarly, access to health services can be limited by poor people’s low self-esteem and lack of self-confidence [27]. Consistent with our study findings, some Ghanaian women had to tolerate being rebuked and treated disrespectfully by healthcare workers without speaking out in order to access care in maternity wards [56].

One of the strengths of the study was its focus on people in the lowest wealth quintile of whom pro-poor health financing is meant to benefit the most. Secondly, various frameworks have been developed to elaborate the dimensions of access [16, 27, 69], however, our study findings contribute to this literature by showing that for population groups that rely heavily on free health care services, healthcare worker strikes are an important access barrier under the availability dimension while discrimination by healthcare workers and absence of effective grievance redress mechanisms are key access barriers under the acceptability dimension. One of the limitations of this study is that the findings may not be generalizable. However, the findings contribute to the literature on access barriers that hinder pro-poor health financing initiatives from enhancing access to care for the poor in similar LMICs. Another limitation is that we did not interview people in wealthier quintiles. Though this was beyond the scope of this study, it would have strengthened existing evidence on how experiences with health financing mechanisms differ across various wealth quintiles.

### Policy implications

Our findings support literature that shows that, for pro-poor health financing policies to be successful in enhancing access to care across the four access dimensions, they must be implemented hand in hand with policies that strengthen health service delivery systems and structures [1]. Since health service delivery has been devolved to the counties, we make the following recommendations on how county governments can enhance the effectiveness of health financing reforms that target the poor in Kenya. To enhance geographical accessibility to healthcare services there is a need to improve the network of health facilities and the condition of roads with a specific focus on rural and marginalized areas where the poor reside predominantly. Increasing geographical access will also contribute to reducing the burden of transport costs. An additional policy option that could be considered is the incorporation of transport vouchers in the design of pro-poor health financing mechanisms. To ensure availability of care, there is need to strengthen the capacity of public health facilities to offer quality health services by improving inputs such as human resources for health, drugs, medical supplies, and equipment. To enhance financial risk protection for the poor, monitoring and accountability mechanisms of free healthcare initiatives should be strengthened to ensure strict adherence to the policies and to eliminate user fees at PHC facilities and informal payments for health services. This could entail the county governments and the NHIF improving supportive supervision to health facilities and increasing awareness on the services that the poor are entitled to under pro-poor health financing reforms. In addition, there is a need for increased and stable financial support from county governments to public healthcare facilities to facilitate service provision.

To improve the acceptability of care, county governments, and the NHIF should ensure the availability of effective complaints and client feedback mechanisms, and ensure that the poor and other vulnerable groups are empowered to engage in public participation. County governments should also collaborate with health facility managers to nurture good healthcare provider-patient relationships that improve health system responsiveness to the needs of the poor and vulnerable by eliminating discrimination and disrespectful treatment. Lastly, continuous quality improvement measures should be established within health facilities to evaluate the delivery and quality of health services provided under pro-poor health financing reforms. The latter should be accompanied by operational research to enable measurement of outcomes of quality improvement interventions to further inform decision making.

## Conclusions

In Kenya, health financing reforms aimed at enhancing financial risk protection for the poor such as user fee removal in PHC facilities, free maternity services, and HISP improved access to healthcare services to some extent. However, our study findings reveal that healthcare access barriers persist among the poor in Kenya despite the existence of health financing reforms that target them. Therefore, to enhance the effectiveness of these pro-poor reforms and to ensure that the poor in Kenya and other LMICs are not left behind, there is a need to address barriers to care across all access dimensions including geographical accessibility, availability, and acceptability.

## Data Availability

All the data for this study is available and it can be accessed at the KEMRI-Wellcome Trust Research Programme, subject to institutional data governance committee policies.

## Abbreviations

FGD: Focus Group Discussion
HDSS: Health and Demographic Surveillance System
HISP: Health Insurance Subsidy Programme
IDI: In-depth Interview
KEMRI-CDC: Kenya Medical Research Institute-Center for Disease Control and Prevention
LMIC: Low and Middle-Income Countries
NUHDSS: Nairobi Urban Health and Demographic Surveillance System
OOP: Out of Pocket Payment
PHC: Primary Health Care
THE: Total Health Expenditure
UHC: Universal Health Coverage
